# Forced Biomineralization: A Review

**DOI:** 10.3390/biomimetics6030046

**Published:** 2021-07-12

**Authors:** Hermann Ehrlich, Elizabeth Bailey, Marcin Wysokowski, Teofil Jesionowski

**Affiliations:** 1 Institute of Electronic and Sensor Materials, TU Bergakademie Freiberg, 09599 Freiberg, Germany; 2 Center for Advanced Technology, Adam Mickiewicz University, 61614 Poznan, Poland; 3 Centre for Climate Change Research, Toronto, ON M4P 1J4, Canada; 4 ICUBE-University of Toronto Mississauga, Mississauga, ON L5L 1C6, Canada; 5 Department of Astronomy and Astrophysics, University of California, Santa Cruz, CA 95064, USA; lizbailey@ucsc.edu; 6 Faculty of Chemical Technology, Institute of Chemical Technology and Engineering, Poznan University of Technology, 60-965 Poznan, Poland

**Keywords:** biomineralization, polyextremophiles, extreme environments, extreme biomimetics

## Abstract

Biologically induced and controlled mineralization of metals promotes the development of protective structures to shield cells from thermal, chemical, and ultraviolet stresses. Metal biomineralization is widely considered to have been relevant for the survival of life in the environmental conditions of ancient terrestrial oceans. Similar behavior is seen among extremophilic biomineralizers today, which have evolved to inhabit a variety of industrial aqueous environments with elevated metal concentrations. As an example of extreme biomineralization, we introduce the category of “forced biomineralization”, which we use to refer to the biologically mediated sequestration of dissolved metals and metalloids into minerals. We discuss forced mineralization as it is known to be carried out by a variety of organisms, including polyextremophiles in a range of psychrophilic, thermophilic, anaerobic, alkaliphilic, acidophilic, and halophilic conditions, as well as in environments with very high or toxic metal ion concentrations. While much additional work lies ahead to characterize the various pathways by which these biominerals form, forced biomineralization has been shown to provide insights for the progression of extreme biomimetics, allowing for promising new forays into creating the next generation of composites using organic-templating approaches under biologically extreme laboratory conditions relevant to a wide range of industrial conditions.

## 1. Introduction

The modern study of biomineralogy represents an interdisciplinary research field dealing with the ability of life to form minerals through biologically mediated processes. A special focus has centered on understanding fundamental mechanisms underlying the biological production of minerals, as well as the fossil preservation of these minerals during the billions of years of evolution of biomineralizers, which have included both uni- and multicellular organisms [1,2,3,4,5,6]. Our review of numerous recent scientific publications on biomineralization has revealed emphasis of the overall scientific attention towards calcification [7,8,9,10,11,12,13], biosilicification [14,15,16], biomagnetism [17,18], and multiphase biomineralization [19,20,21,22,23]. Traditional objects of study continue to include molluscs [24,25,26,27,28], sea urchins [29], and skeletal structures such as eggshells [30], teeth [31,32], and bones [33,34,35,36]. While it is understandable to focus on the biomineralizing organisms typically encountered by humans on Earth, this focus tends to overlook a variety of distinct biomineralization pathways seen in extremophiles. As discussed in this work, an understanding of the varied pathways for the biologically mediated production of minerals, including metals, offers biomimetic avenues for the production of economically relevant materials and appears to hold the key to understanding the fossil record of early life.

It is well established [37] that the phenomenon known as “biologically induced” mineralization is based on the secondary precipitation of minerals occurring as a result of interactions between biological activity and the environment, where cell surfaces often act as causative agents for nucleation and subsequent mineral growth [38]. Here, we propose the introduction of the term “*forced biomineralization*” to refer to the special case for induced biomineralization when a high concentration of metallic ions leads to the development of diverse biomineralized structures contributing to the survival of extremophiles. This article examines current views on how biomineralization processes have been employed by organisms under extreme environmental conditions, such as high concentrations of metal ions like Au, Zn, Mn, Cr, Ni, V, Fe, and metalloid As. In this review, we will discuss a variety of organisms that sequester metals into biominerals. These organisms have often been found living in the toxic waste pools and runoff areas of mines. While--following the convention of other extremophiles–one might call these organisms a name such as “metallophiles,” the current view is that many of these organisms do not “love” (“-phile”; Greek) toxic metals, so much as tolerate them. In many of the cases we will discuss, forced biomineralization is thought to be a common way for life to cope with environments with high metal concentrations. These forced biominerals tend to have less morphological complexity than, for example, the complex biomineralized spikes and shields developed around the time of the Cambrian explosion. Yet, the pathways of forced biomineralization can offer a window into biological strategies for coping with toxic environments. 

This review article discusses a wide variety of extremophiles that exhibit forced biomineralization. They range from single-celled prokaryotic and eukaryotic microorganisms to various self-organizing multicellular forms such as microbial mats and metazoans. While organisms exhibiting forced biomineralization tend to represent Life’s more recent evolutionary forays into modern extreme (typically industrialized) environments, an understanding of forced biomineralization may offer a window into the types of strategies used by life in special environmental conditions widely considered to be relevant to the origin of life (i.e., areas of hydrothermal activity). Understanding of the processes behind forced biomineralization may aid in the future mitigation of industrial disasters involving metal contamination, or alternatively, stimulate the development of novel biominerals and composite materials that incorporate organisms’ abilities to accumulate metals–including metals less commonly encountered in nature, such as Europium [39]. 

## 2. Biomineralization of Gold

**Figure 1:**
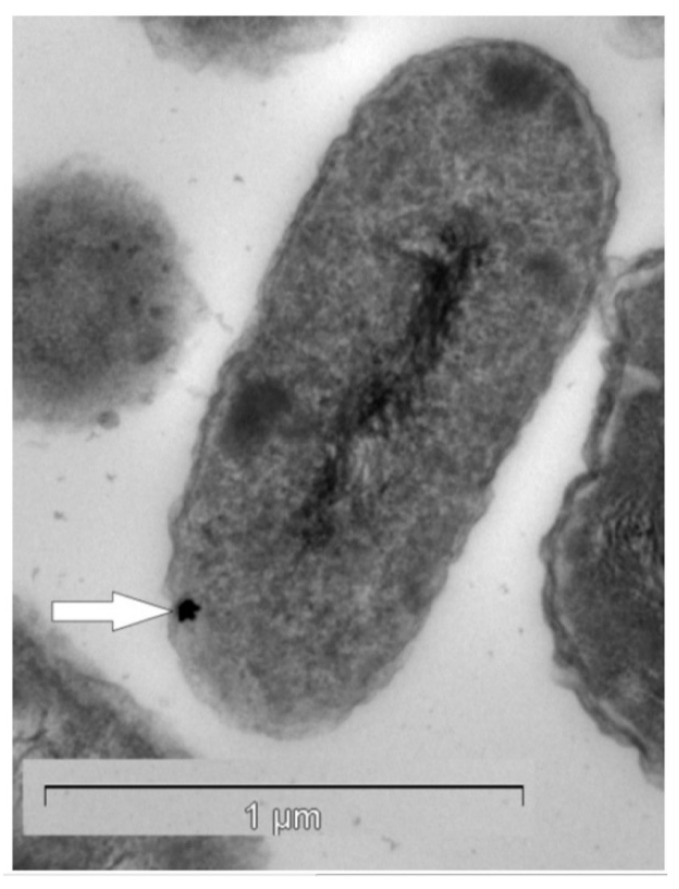
Transmission electron micrograph of a *Cupriavidus metallidurans* ultra-thin section containing a gold nano-particle (white arrow) in the periplasm. Reproduced from [53] with permission of PNAS, Copyright 2009 National Academy of Sciences.

Gold of microbial origin, which occurs in nano-particulate, spheroidal, and bacteriomorphic forms [40,41,42,43], has been reported as a biological response to highly toxic gold-complexes [44]. Both the geomicrobiology and the biogeochemistry of gold have been described by Reith et al. [40,41] and Southam et al. [42], respectively. Microbially originated gold is found in a variety of environmental niches, including those with very low gold concentrations [45,46]. Gold does not form free ions in aqueous solution at surface conditions but rather occurs as metallic nano-particles (0), as well as aurous (I) and auric (III) complexes [47]. Biochemical responses to gold have been studied in numerous bacterial strains, especially in *Cupriavidus metallidurans* and *Delftia acidovorans*, which harbor the ability to withstand and accumulate high levels of gold (Figure 1) (for details, see [48,49,50]. For example, the process of gold detoxification in *C. metallidurans* is a complex phenomenon where several gene clusters, involved in metal resistance (cop, cup, ars, mer) and gold-specific operon (gig), for gold-induced genes are involved. 

Furthermore, the combination of efflux, reduction, and methylation of gold-complexes, leading to the formation of gold (I)-C-compounds and metallic nano-particulate gold, have been suggested [49]. It was also proposed that gene-regulated gold-handling systems are involved in three processes: the uptake of gold complexes into the cytoplasm, the export of gold (I) back to the periplasm after reduction, and further chemical reduction to gold (0) in the periplasm [49]. Gold biomineralization occurs in strains of *C. metallidurans*. These strains either show planctonic behavior [48] or reside in sheet-like biofilms [51,52]. The gold formed on exopolymeric layers within biofilms was found to take on several morphologies, including isolated nanoparticles, conglomerates of nano-particles directly associated with cells, and larger (> 1 µm) extracellular rod-shaped, hollow spheroidal, and framboidal particles [52].

**Figure 2:**
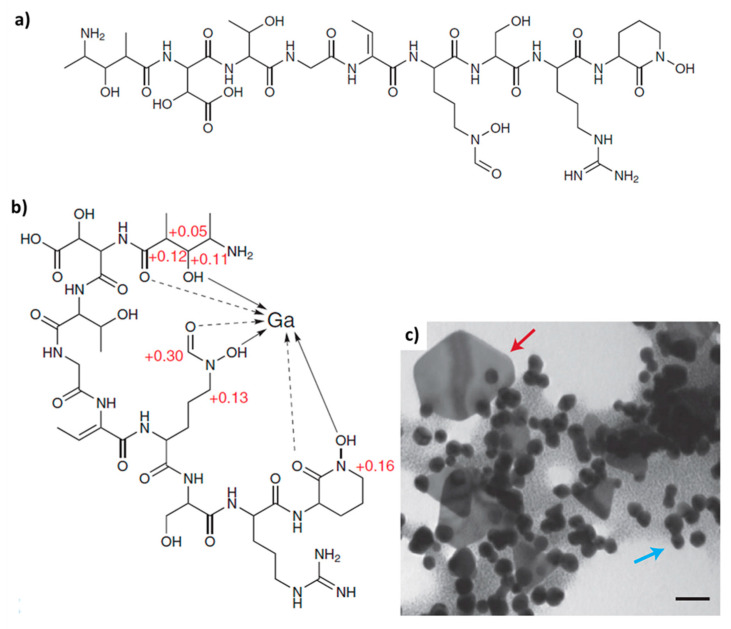
(**a**) Structure of *defltibactin A*, (**b**) Gallium NMR confirms that delftibactin has a single metal-binding site. (**c**) TEM of delftibactin–gold (2:1) complex after 10 min reveals the presence of colloidal and octahedral gold nanoparticles, reminiscent of those seen in natural deposits. Blue arrow, colloidal gold. Red arrow, octahedral gold. (Scale bar 50 nm). Reprinted with permission from Macmillan Publishers Ltd: Nature Chemical Biology [55], copyright 2013.

Various biomacromolecules have been reported to show templating activity with respect to gold biomineralization in different microorganisms [54]. For example, the non-ribosomal peptide of *Delftia acidovorans*, termed *delftibactin A* (Figure 2a), is responsible for chelating soluble Au^3+^ and directly precipitating it as a complex, or by binding and reducing gold through oxidative decarboxylation before chelating a second Au^3+^ ion and precipitating a complex (Figure 2c) [55]. Delftibactin seems to be the first known case of an evolutionarily co-opted metallophore that protects its producer from toxic soluble gold and provides a mechanism for bacterial gold biomineralization.

Some cells of a Gram-negative, non-spore-forming, and metal resistant ß-proteobacterium *Ralstonia metallidurans* displayed no discrete areas of gold accumulation and appeared to be entirely covered by gold, which seems to be associated with sulfur- and phosphorus-containing substances in the cell membrane or the cell wall [48]. In the cyanobacterium *Phormidium boryanum,* biogenic synthesis of secondary octahedral gold crystals from gold (III)-chloride solution occurs via an amorphous gold (I)-sulfide intermediate [56,57]. The single-step biological synthesis of polycrystalline gold nanotriangles, using the extract of endophytic actinomycetes *Saccharomonospora* sp.; has been reported by Verma et al. [58]. However, the exact mechanism for this shape-oriented synthesis of nanostructured gold remains unclear. Gold ions bind on the oppositely charged mycelia of fungus *Rhizopus oryzae* through electrostatic interaction with phosphoproteins and are then reduced to Au (I) species due to the high redox potential of Au (III) [59,60].

Exopolysaccharides exhibit similar behavior. A recent study by Ravendraan and co-workers [60] addresses potential applications of the substance *mauran*, a bacterial-sulfated exopolysaccharide extracted from halophilic *Halomonas maura*, for the reduction and stabilization of Au-nanoparticles [60]. Metal ion reduction and gold nanoparticle stabilization are also believed to occur by an enzymatic process [61]. This process is also observed in an alkalothermophilic actinomycete *Thermomonospora* sp., which has optimum growth at pH 9 and 50 °C [53]. 

As reported by Jones et al. [62], enzymatically catalyzed precipitation of gold has also been observed in thermophilic and hyperthermophilic bacteria and archaea (e.g., *Thermotoga maritime*, *Pyrobaculum islandicum*) isolated from hot spring systems in New Zealand. In this case, biogenic gold was forming in hot anaerobic spring-waters (at ca. 75 °C). Thermophilic microorganisms, in particular, seem to be an appreciable source of bioinspiration for designing new materials through approaches in extreme biomimetics. For example, a novel approach was developed for preparing a composite biomaterial that consists of gold nanoparticles trapped within an unfolded protein, the bovine serum albumin (BSA) fiber [63]. In general, the formation of secondary gold nano- and micro-grains via biomineralization, using diverse microorganisms, has stimulated the development of gold-based biotechnology [64,65,66,67]. Biomineralized gold nanoparticles have been considered as a potentially useful antitumor agent [68].

Another potential field of application is related to the antimicrobial activity of gold nanoparticles, especially against pathogens resistant to multiple antibiotics (for review, see [69,70]). It was demonstrated that the catalytic and antibacterial mechanism of gold nanoparticles increases with a decrease in average size [67]. In laboratory experiments, it was also shown that gold nanoparticles generate ‘holes’ in bacterial cell walls, thereby increasing permeability, resulting in the leakage of cell contents and eventual cell death [70]. However, this leaves us with a controversy: given that certain bacteria produce nanoparticulate gold under specific environmental conditions, have these organisms evolved special resistance to the antibacterial activity seen by gold nanoparticles? Or are the nanoparticles produced by these organisms merely a “lesser of two evils,” such that the bacterially mediated precipitation of gold into nano-nuggets is still damaging to cell walls but favorable for survival due to the reduction of concentrations?

We would like to conclude this section with an optimistic note for biomaterials scientists from a recent publication in Nature: “*Deeper understanding of the link between bacteria and gold could even lead to bacteria producing customized gold nuggets*” [71,72]. Thus, good luck!

## 3. Bioscorodite

The metalloid arsenic (As) can form inorganic (As (V), arsenate; As (III), arsenite; As (0), As (−III), arsine; As (−I) in arsenopyrite; and As (+II) in realgar) and organic (i.e., methylated) compounds (for review, see [73]). Being more mobile in the environment, As (III) is listed [73] as 25 to 60 times more toxic than As (V). Examples of extreme biomineralization of As with respect to the formation of thermodynamically more stable phases are also known. 

The identification of several arsenic-sulfide minerals formed during microbial reduction of arsenate As (V) and sulfate (SO_4_^2−^) have been reported [74]. For example, an anaerobic moderately thermophilic arsenic-reducing bacterium closely related to the *Caloramator* and *Thermobrachium* species and proposed as strain YeAs [75] produces an arsenic sulfide mineral identified as ß-realgar (ß-AsS). A summary of the reactions involved in the extreme biomineralization of arsenate to arsenic sulfides has been proposed by Rodriguez-Freire [74]. 

Ethanol acetogenesis:

CH_3_CH_2_OH + H_2_O → CH_3_COO^−^ + 2 H_2_ + H^+^ (1)

Acetoclastic methanogenesis:

CH_3_COOH → CH_4_ + CO_2_ (2)

Hydrogenotrophic methanogenesis:

4 H_2_ + CO_2_ → CH_4_ + 2 H_2_O (3)

Sulfate reduction coupled to H_2_ oxidation:

SO_4_^2−^ + 4 H_2_ +2 H^+^ → H_2_S + 4 H_2_O (4)

Arsenate reduction coupled to H_2_ oxidation:

H_2_AsO_4_^−^ + H_2_ + H^+^ → H_3_AsO_3_ + 2 H_2_O (5)

Mineralization:

*x* H_3_AsO_4_^−^ + *y* HS^−^ + (3*x* − *y*) H^+^ → As*_x_*S*_y_*↓ + 3*x* H_2_O (6)

When: *x* = *y* = 1 − realgar (AsS) formation

*x* = 2; *y* = 3 − orpiment (As_2_S_3_) formation

The arsenic-respiring bacterium *Desulfosporosinus auripigmenti* precipitates monodisperse spherical As_2_S_3_ particles, both intra- and extracellularly, under sulfate-reducing conditions [76]. The anaerobic *Shewanella* sp. strain HN produce an extracellular network of filamentous arsenic-sulfide (As-S) nanotubes in the presence of As(V) and S_2_O_3_^2−^. These arsenic-sulfide (As-S) nanotubes (20 to 100 nm diameter, 30 µm length) were initially amorphous As_2_S_3_ but evolved with increasing incubation time toward polycrystalline phases of the chalcogenide minerals realgar (AsS) and duranusite (As_4_S) [77].

One industrially relevant example of As-related biomineralization under extreme conditions is bioscorodite (FeAsO_4_·2H_2_O), for which microbial crystallization was demonstrated by Gonzalez-Contreras et al. [78,79,80] using As(V) as a reactant. Bioscorodite is precipitated in one single step at pH 1.2 and 70 °C. Batch crystallization of bioscorodite leads to agglomeration of precipitates and formation of flakes; scaling of bioscorodite precipitates was also observed in continually stirred tank reactors. The term “indirect biomineralization of scorodite” was proposed in 2012 and was patented by Paques B.V. (Balk, The Netherlands) as the ARSENOTEQ™ process [79,80]. According to the proposed biotechnological approach, the iron-oxidizing archaeon *Acidianus suljidivorans* is able to precipitate scorodite in the absence of any primary minerals or seed crystals when grown on 0.7 g/L ferrous iron (Fe^2+^) at 80 °C and pH 0.8 in the presence of 1.9 g/L arsenate (H_3_AsO_4_). 

Scorodite biomineral formation has also been noted by the acidophilic iron-oxidizing *Sulfolobales* spp. at 75 °C and a pH of 1 [78,81]. It was suggested that the mechanism of scorodite formation in this microorganism begins with the sorption of ferric iron and arsenate onto the cell surface, followed by the formation of ferric arsenate nuclei from the adsorbed metal species. Finally, partial encrustation of the cells was observed. Formation of bioscorodite has been reported in thermo-acidophilic Fe(II)-oxidizing archaeon *Ac. brierleyi* under specific cultivation conditions as well, with an optimal pH of 1.5 to 2.0 and temperature of 70 °C [82,83]. It was shown that bioscorodite can still be crystallized in the presence of Cu(II) by feeding scorodite seeds from synthetic copper-refining As(III)-bearing wastewaters [83].

The advantages of extreme biotechnology with respect to bioscorodite formation are summarized by Gonzalez-Contreras [80] as follows:

- “ the bioscorodite crystal features are very similar to the mineral;
- supersaturation is controlled on a microscale by biological iron oxidation at 80 °C without the use of seed material;
- the biological oxidation does not need the use of strong chemical oxidants;
- arsenic levels of at least 1g/L can be treated (potential future applications of bioscorodite crystallization for metallurgical streams);
- crystal and agglomerates size enable an efficient solid-liquid separation.”

In spite of the progress of research with respect to bioscorodite [84,85], there remains a lack of information about the role of organic matrices in the formation of biogenic scorodite under the very specific conditions listed above. An understanding of this problem may hold critical advances to the problem of arsenic separation from water.

## 4. Biogenic Hydrozincite

The toxic impacts of Zn ions can vary according to various factors, including high pH or low water hardness. Depending on water hardness, the LC_50_ (50% lethal concentration) of Zn^2+^ for *Capoeta fusca* fish is 13.7, 74.4, and 102.9 mg/L for soft, medium, and hard water respectively [86]. Eisler [87] proposed aquatic life protection criteria to include mean Zn concentrations of 47 to 59 µg/L in freshwater and 58 to 86 µg/L in seawater. However, in some extreme environments–such as the Iron Mountains, California, or Sepetiba Bay, Brazil– the concentration of zinc can reach grams per liter (see review [88]). Forced biomineralization with respect to Zn leads to the development of specific biomineralized structures (globules, shells) that likely help to prevent cell entombment. 

A typical example of forced Zn biomineralization, leading to the formation of hydrozincite, Zn_5_(CO_3_)_2_(OH)_6_ (for structural details see [89]) as well as the Zn-bearing amorphous biomineral, is a unique phenomenon occurring along the Naracauli stream, Sardinia [88,89,90]. Due to local mining activities, the maximum Zn concentration in waters from this area attains several hundred mg per liter [91,92]. The amorphous white Zn biomineral shows a local atomic environment that may be compatible with a long-range order of zinc silicate (hemimorphite, Zn_4_(Si_2_O_7_)(OH)_2_·H_2_O). It was shown [93] that the formation of hydrozincite is mediated by a photosynthetic community composed of a single-cell photosynthetic microalga (*Chlorella* sp.; Chlorophyta) and a cyanobacterium (*Scytonema* sp.). However, the precipitation of the amorphous Zn phase is associated with a cyanobacterium identified as *Leptolyngbya frigida*. The biomineralization of hydrozincite and Zn-silicate is possible due to long-term adaptations of the microorganism community to prevailing hydrological, geochemical, and mineralogical conditions imposed by the water chemistry of the Naracauli stream [94]. The mechanism governing the formation of these Zn-based biominerals at the molecular level remains unclear. It is suggested that hydrozincite biomineralization is an example of epicellular biomineralization [93], where electronegatively charged extracellular polysaccharides show templating activity [95,96]. 

The biological driver of this kind of Zn biomineralization is probably based on the ability of photosynthetic microorganisms to use hydrozincite to shield themselves from UV radiation–and in the process, decrease Zn concentrations and increase their chances of survival [94]. It was proposed [91] that the CO_2_ fixation from dissolved HCO_3_^−^ and release of OH^−^ during photosynthesis leads to a shift in the carbonate species equilibrium, and consequently, to a local oversaturation with respect to hydrozincite, around the surface of the microorganisms. The following reactions were proposed:

HCO_3_^−^ + H_2_O ↔ (CH_2_O) + OH^−^ + O_2_ (photosynthesis) (7)

HCO_3_^−^ + OH^−^ ↔ CO_3_^2−^ + H_2_O (8)

2 CO_3_^2−^ + 5 Zn^2+^ + 6 H_2_O ↔ Zn_5_(CO_3_)_2_(OH)_6_ + 6 H^+^ (9)

The hierarchical organization of hydrozincite is well-studied using different electron microscopy techniques [91,93,97]. Briefly, the spherical precipitates of hydrozincite biomineral are made from nanocrystals, aggregated with an imperfect orientation. These nanocrystalline aggregates form mesocrystals, about 100 nm thick platelets flattened onto the (100) crystal surface. In turn, the mesocrystals aggregate to form globules and sheaths all around the extracellular organic matrices on the surface of these microorganisms. These globules merge into each other as they grow and appear to maintain a smooth texture, with the porous structure only appearing at later stages of growth [98].

Spherical Zn-containing minerals that form aggregates up to 10 µm in diameter have been observed within natural biofilms dominated by relatively aerotolerant sulfate-reducing bacteria of the family *Desulfobacteriaceae*, in a flooded tunnel within carbonate rocks that host the Piquette Pb-Zn deposit (Tennyson, Wisconsin) [99]. However, in this case, spherical aggregates of 2 to 5 nm in diameter have been represented by sphalerite (ZnS). Zinc concentrations in the biofilm were measured at about 10^6^ times that of the associated groundwater (0.09 to 1.1 ppm zinc). These results demonstrate that coupled geochemical and microbial processes can efficiently strip Zn from solutions with Zn contents < 1 ppm [97]. The phenomenon described in *Science* (2000) is now used for biotechnological aims. As recently reported [100], microbially mediated zinc sulfide nanoparticles were manufactured in large amounts using modern pilot-plant scale reactors.

Zinc-related forced biomineralization in metazoans has also been characterized. Examples include molluscs grown in mine-polluted seabed sediments [101], as well as in *Alvinella pompejana* (Terebellida: Annelida) worms, which are typical representatives of annelids in hydrothermal vent fauna communities. Nanocrystalline zinc-iron sulphide minerals with the composition (Zn_0.88_Fe_0.12_)S were found within the exoskeletons of *A. pompejana* [1] collected at 9° N on the East Pacific Rise. The nanocrystals of this sphalerite-like biomineral are grouped in submicrometer-sized clusters, which form nanolayers concentrically to the proteinaceous tube axis. Thus, this biomineral represents the unique example of zinc-iron biologically induced mineralization in metazoans that survive under harsh environmental conditions.

## 5. Biogenic Manganese Oxides

Mn(II)-oxidizing bacteria and fungi are key players in ancient and modern biogeochemical environments. Mn(II)-oxidizing microorganisms possess the ability to catalyze the oxidation of divalent, soluble Mn(II) to insoluble manganese oxides of the general formula MnO_x_ (where x = 1, 2). They are ubiquitous in nature and are well investigated and described, including possible mechanisms of the biomineral formation, in numerous review papers [102,103,104,105,106,107,108]. Diverse Mn(II)-oxidizing organisms produce nanoorganized structures called Mn(IV) bio-oxides [105], biogenic manganese oxides (BioMnO_x_) [109], or manganese oxide biominerals [110] (see Table 1); with a broad variety of morphologies such as biomineralized sheaths, globules, lamellas or nanonodules [111].

These have been studied in detail using different electron microscopy techniques. Microbially determined Mn(II) oxidation was found in habitats where Mn can reach toxic levels, and therefore, it is suggested that biogenic Mn-containing biominerals may serve to protect cells from Mn toxicity or UV radiation [112]. The encrusted Mn oxides, which may be acting as a protective barrier from toxic metal ions, have been found not only on cells but also on spores and spore coats of numerous bacteria [113,114,115,116]. Recently, molecular studies with marine *Bacillus* spores have identified the mnx (Mn oxidation) genes, including mnxG, encoding a putative multicopper oxidase, as responsible for unique two-electron oxidation [117].

**Table 1:** Diversity of biogenic manganese oxides.

Biomineral Name	Chemical Composition	Reference
Switzerite	(Mn, Fe)_3_(PO_4_)_2_	[118]
Bixbyite	(Mn, Fe)_2_O_3_	[118]
Hausmannite	Mn^2+^Mn^4+^_2_O_4_	[111,118]
Pyrolusite	MnO_2_	[118]
Manganosite	MnO	[118]
Romanechite	(Ba, H_2_O)_2_(Mn^4+^, Mn^3+^)_5_O_10_	[119]
Rhodochrosite	MnCO_3_	[118]
Todorokite	Mn_4_O_7_ H_2_O	[120,121]
Birnessite	Na_4_Mn_14_O_27_ 9 H_2_O	[122,123]
Bixybyite-like	Mn_2_O_3_	[124]

Although the biomineralogy of the biogenic manganese oxide is ultimately dependent on physico-chemical conditions, we still have limited knowledge about the formation of biogenic Mn oxides in extremophiles. For example, there are only a few species of *Streptomyces* and *Cephalosportium,* as well as some acidophilic microbial communities, which are known to be able to produce these kinds of oxides at pH levels between 4.8 and 5.5 (for review, see [125]). However, some microalgae are able to carry out Mn-biomineralization at a lower pH. An acid-tolerant microalga, *Chlamydomonas* sp., was isolated and enriched from a mat surrounding a drainage ditch with approximately 8 mg/L of Mn^2+^ at pH 2.1 [126].

Other extremophiles are related, not to acidophilic but, to thermophilic microorganisms. Thermophilic *Caldimonas manganoxidans* strain HST isolated by Takeda and co-workers from hot spring was an aerobic chemo-organotrophic bacterium with an optimum growth temperature of 50 °C and the ability to produce biogenic manganese oxides under laboratory conditions [127]. As reported by Dick et al. [128], marine Mn (II)-oxidizing *Bacillus* sp. isolated from hydrothermal vent sediments may be able to grow in some moderately hot sediments. However, they were most likely only present as spores at temperatures above 60 °C. 

Recently, it was hypothesized [129] that Mn biominerals, which are widespread in the environment, could be used for the synthesis of new electrode materials.

## 6. Biogenic Nickel Minerals

The nickel homeostasis processes used by microorganisms are still under study (see review [130,131], and much is left to learn about Ni-based biomineralization in both mesophile and extremophile communities. Only a few examples can be found in the literature [132,133].

The formation of a new biomineral, identified as Ni-struvite (Ni(NH_4_)(PO_4_) 6 H_2_O), using a nickel resistant E13 strain [131,132] has been described by Haferburg and co-workers [133]. This microorganism was isolated from the former uranium mining area near Ronneburg, Eastern Thuringia, Germany, where former mining activities have resulted in nickel concentrations of up to 30 mM. It is postulated that the capacity to induce this kind of forced biomineralization with respect to the formation of the nickel-containing biomineral constitutes a resistance factor allowing the soil microorganism to withstand high nickel concentrations [133].

One strain of *Pseudomonas aeruginosa* was reported, which accumulated nickel in phosphide (Ni_5_P_4_, NiP_2,_ and Ni_12_P_5_) and carbide (Ni_3_C) crystals, mostly in the cell envelope region. Thus, 88% of the accumulated nickel was restricted to the periplasm and membrane [134].

## 7. Biogenic Vanadate

In spite of more than 200 reported minerals in which vanadium occurs in different oxidation states [135,136], and despite the presence of concentrated vanadium in various industrial and mining processes, vanadium-containing biominerals are rare. Pentavalent vanadium is the more soluble compound and the most toxic form [137]. Some cyanobacteria (*Nostoc puncteforme* strain N467, *Phormidium laminosum* strain N17), as well as bacteria (*Pseudomonas isachenkovii* and *Pseudomonas vanadium-reductans*), can tolerate high concentrations of vanadium in corresponding natural environments [138]. These bacterial strains are capable of reducing pentavalent vanadium under anaerobic conditions at pH 8 and at concentrations below their respective limits of tolerance. For example, *P. isachenkovii* tolerated concentrations of pentavalent V greater than 6 g/L. In this study, vanadate was reduced to tetra- and trivalent states by growing cultures with organic electron donors, as well as with molecular hydrogen and carbon monoxide. Finally, sherwoodite-like (Ca_9_Al_2_V^5+^_4_V^5+^_24_O_80_∙56 H_2_O) biominerals have been identified on the surface of bacterial cells [138]. More recently, microbial reduction of vanadate (V^5+^) by a mesophilic (*Methanosarcina mazei,* optimal temperature 37 °C) and a thermophilic (*Methanothermobacter thermautotrophicus*, optimum temperature 65 °C) methanogen was studied by Zhang et al. [139]. Both archeans reduced up to 10 mM and 5 mM of V^5+^ respectively in a growth medium. Although the V^5+^ bioreduction occurred extracellularly and resulted in concomitant precipitation of an amorphous V(IV) solid, this biomineral was not previously identified or characterized.

Recently, it was reported that such basidiomycete fungi species as *Amanita muscaria*, *Armillaria cepistipes*, *Xerocomus badius,* and *Bjerkandera adusta* were able to accumulate vanadium from VOSO_4_ and NaVO_3_ medium up to 51.3 mg g^−1^ [140]. However, it is not clear what specific types of V-based biomineral phases can be achieved using these microorganisms. In contrast, a metal-reducing bacterium such as *Geobacter sulfurreducens* is able to produce biogenic nanoscale vanadium magnetite by converting V(V)-bearing ferrihydrites through corresponding reductive transformation [141]. 

## 8. Biogenic Chromium Minerals

Hexavalent chromium is generally found to be a particularly toxic ion [142]. Chromium-resistant bacteria, and the mechanisms of chromium detoxification based on chromate reductase activity, have been recently described in detail in the reviews by Narayani & Vidya Shetty [143], Thatoi et al. [144], and Joutey et al. [145]. Intriguingly, some extremophilic microorganisms are also involved in this process. For example, an anaerobic thermophilic bacterium (strain TOR 39) carries out the reduction of Cr(VI) at 65 °C [144,146] and a hyperthermophilic archaea Geothermobacterium ferrireducens at 100 °C [147]. 

An unidentified Cr-containing precipitate has been observed on the surface of *Thiobacillus ferrooxidans* cells that were able to tolerate Cr^3+^ concentrations up to 75 mM during growth on ferrous sulphate at pH 1.4 [142]. Furthermore, unidentified intracellular chromium-containing aggregates have been observed using electron microscopy in marine *Roseobacter* YSCB strains [148].

Recently, a method for biotransformation of toxic Cr(VI) ions into Cr_2_O_3_ nanoparticles by the yeast *Schwanniomyces occidentalis* has been reported [149]. Unfortunately, the formation of these biogenic nanoparticles, sized between ~10 and 60 nm, has yet to be analyzed from the biomineralogical point of view. A similar situation has been observed with root nodule bacterium *Sinorhizobium* sp. SAR1 that tolerated Cr concentrations up to 1 mM due to the production of exopolymers [150].

**Figure 3:**
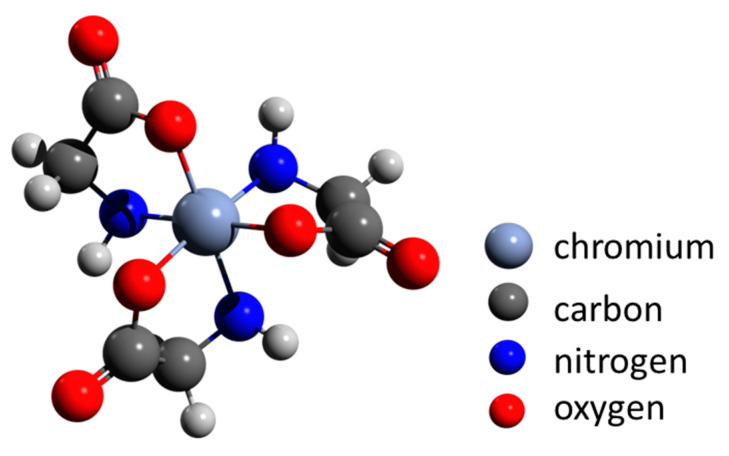
The chemical structure of glycine-Cr(III) as a proposed precursor for hydrothermal crystallization of α-CrOOH (grimaldiite) and β-CrOOH (guyanaite) at 250 °C. Adapted from Cheng et al. [151].

Cheng and co-workers [151] proposed that biomineralization is an environmentally important issue in the remediation of heavy-metal contamination, including chromium. They described transformation from organo-Cr(III) to trivalent chromium minerals (guyanaite/grimaldiite) by hydrothermal treatment at 200 °C to simulate geothermal conditions. The authors show that amorphous complexes of glycine-Cr(III) are stable up to 150 °C. Heating up to 250 °C for 7 days results in the formation of α-CrOOH (grimaldiite) and layered β-CrOOH (guyanaite) (Figure 3) crystals, 10 to 20 nm long and 2 to 3 nm wide. These results suggest that naturally occurring amorphous organo-Cr(III) can be converted into minerals consisting of nanosheets under certain environmental conditions, such as those in a volcanic eruption or during geothermal activity. Such reactions may be a model for bioremediation of pollution by soluble chromium.

## 9. Iron Biomineralization and Extremophilic Organisms

Varied forms of iron-containing biominerals have been discovered in both pro- and eukaryotic organisms, including humans. These biominerals can be synthesized intracellularly [152,153,154,155,156,157,158], extracellularly [158,159,160], and are also found surrounding bacterial adhesive stalks [161]. The strong influence of microbial activity on mineralogical diversity of iron-containing minerals through extracellular and intracellular biomineralization was recently shown [161]. Although Fe-encrusted biomineral structures produced by certain species of Fe-depositing bacteria have been known since the early 19th century (for review see [162,163]), recent work has described numerous species of the iron biomineral *Gallionella*-related stalk-forming iron-oxidizing freshwater bacteria [161] and marine *Zetaproteobacteria* bacteria [164,165,166,167].

The study of iron biomineralization is rapidly advancing [168]. After the classical work by Richard Frankel “*Iron biominerals: an overview*” [169], detailed reviews on microbially and chemically mediated reactions that form the biogeochemical Fe cycle [169,170], iron biomineralization in vertebrates [171] and plants [172], as well as genetic and molecular mechanisms of biomineralizaton [173], including that in magnetotactic bacteria [157] have been recently published. Briefly, the majority of iron-based biominerals are secondary iron sulfides and iron oxides like Fe_2_O_3_ (hematite), FeOOH (goethite), Fe_3_O_4_ (magnetite), green rust (mixed valence hydroxide), Fe_3_(PO_4_)_2_ (vivianite), and FeCO_3_ (siderite) [174]. Hypotheses concerning common ancestry between iron oxide- and iron sulfide-based biomineralization are still under discussion [175]. Some microorganisms, such as *Acidovorax* sp., are able to synthesize several Fe minerals (lepidocrocite, goethite, FePO_4_) simultaneously [176].

J. Kirschvink suggests that Fe_3_O_4_ (magnetite) biomineralization is the most ancient matrix-mediated system; it may have served as the ancestral template for exaptation [177,178]. Consequently, it was not surprising to find reports about the formation of biomagnetite and other iron biominerals in extremophiles and polyextremophiles [179]. Formation of biomagnetite have been reported for: (i) anaerobic [180], (ii) acidophilic [181,182], (iii) alkaliphilic [183], (iv) halophilic [184,185], (v) piezophilic [186,187], (vi) psychrophilic [188,189], and (vii) thermophilic [190,191] microorganisms. For the overview of this specialized topic, we recommend the outstanding paper entitled “*Magnetotactic Bacteria from Extreme Environments*” [192].

Overview of this specialized topic, we recommend the outstanding paper entitled “*Magnetotactic Bacteria from Extreme Environments*” [192].

**Figure 4:**
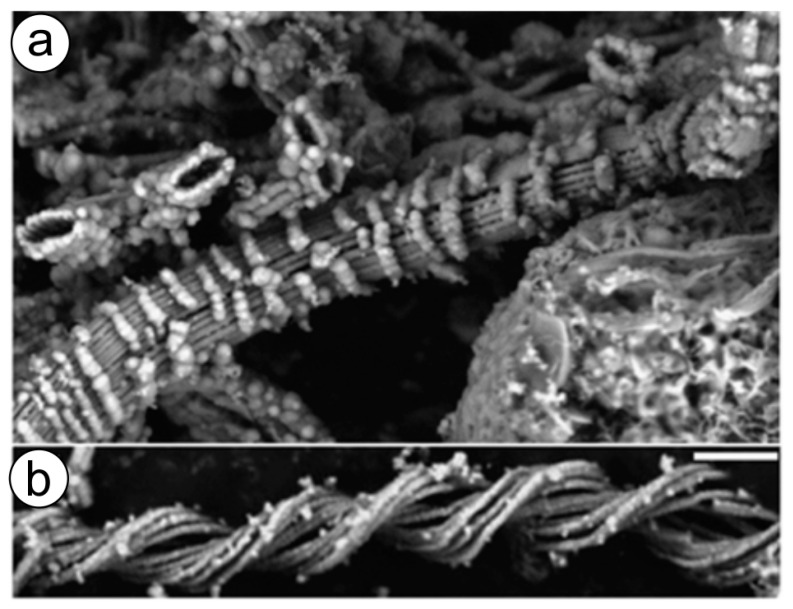
(**a**,**b**) SEM micrographs of mat and crust material from the Ula Nui hydrothermal vent area show diverse Fe-containing structures. The banding patterns of unusual nanoparticle morphologies observed are thought to be of biogenic origin but have not been reported elsewhere. Scale bar 1 µm. (Reprinted by permission from Macmillan Publishers Ltd: The ISME Journal [194], copyright 2011).

Fe oxidizing microorganisms, in the form of microbial mats, live within hot springs [193] as well as near hydrothermal vents (Figure 4) worldwide, where iron concentrations are very high. It was hypothesized that such microbial mats are fed by ultra-diffuse advection of hydrothermal fluids, which derive from a higher-temperature source enriched in Fe, Mn, and Si that has undergone extensive subsurface cooling [194,195].

Microorganisms belonging to hyperthermophilic iron reducers, which are capable of living completely independently of photosynthesis, are found within various hydrothermal environments [167]. Even at temperatures of at least 121 °C, an obligate iron reducer belonging to the Pyrodictiaceae family (Strain 121) was capable of growth in culture [193]. Diverse hyperthermophilic iron reducers, including *Pyrobaculum*, *Geoglobus*, *Ferroglobus*, and *Geothermobacterium* species, produce iron-containing biominerals using H_2_ as the electron donor at temperatures between 85 and 110 °C [174]. *Sulfolobus solfataricus* is an aerobic hyperthermophilic (70 to 90 °C) archaeon that thrives in acidic terrestrial thermal features that are commonly associated with high iron concentrations. Wiedenheft et al. [196] isolated a ~22 kDa protein with little sequence similarity to proteins of known function. The obtained protein shares high sequence similarity with hypothetical proteins in other archaeal and bacterial genomes. Nine of these hypothetical proteins form a monophyletic cluster within the broad superfamily of ferritin-like diiron-carboxylate proteins. By applying higher-order structural predictions and image reconstructions, Wiedenheft et al. [196] indicated that the *S. solfataricus* protein is structurally related to a class of DNA-binding proteins from starved cells; it self-assembles into a hollow dodecameric protein cage having tetrahedral symmetry. The outer shell diameter is ~10 nm, and the interior diameter is ~5 nm. Authors proved through in vitro experiments that the assembled archaeal protein efficiently uses H_2_O_2_ to oxidize Fe(II) to Fe(III) and stores the oxide as a mineral core on the interior surface of the protein cage. The described biomineralization mechanism has been shown to be responsible for the protection of nucleic acids by physically shielding DNA against oxidative damage by consuming the constituents involved in Fenton chemistry [196].

In addition to thermophiles, there are other extremophiles with the capacity to produce iron biominerals under specific conditions. Procaryotic Fe(II) oxidizers are principally divided into the following physiological groups: (i) the acidophilic aerobes, (ii) the neutrophilic aerobes, (iii) the neutrophilic photosynthetic anaerobes, and (iv) the neutrophilic anaerobes dependent on nitrate, perchlorate, or chlorate reduction [197]. 

Examples of iron biomineralization have also been observed in microbial acidophilic communities. ‘Gel-like reddish-brown soft tissue structures’ were formed by marine iron-oxidizing bacteria in hot springs and geothermal areas in Japan [198], growing rapidly and accumulating iron from acidic seawater. It was suggested that after using ferrous ions as the energy source, other acidophilic bacteria could enzymatically oxidize ferrous iron, decreasing the acidity of ambient seawater. Consequently, reactions promoted by photosynthetic bacteria of this microbial community at near-neutral pH led to the formation of solid forms of ferric iron, such as ferrihydrite [198].

The study of iron biomineralization is a growing interdisciplinary area of modern applied science that involves such fields as biotechnology [199,200], nanotechnology [201,202], biomaterial science, and biomedicine [203,204]. Magnetotactic bacteria and magnetosomes have been recently proposed for application in a variety of fields, including nano-scale engineering [205,206], magnetic hyperthermia, magnetic resonance imaging, nucleotide polymorphism detection, and immunoassays [207,208]. One challenging task currently being considered is the use of genetic engineering approaches to transfer the capacity for magnetosome-producing microorganisms to other organisms for the generation of synthetic magnetic living systems for potential industrial-scale biotechnological applications, including medicine, nanotechnology, and the remediation of chemical waste [157].

Knowledge from recently published studies on in vitro artificial diagenesis using Fe (II)-oxidizing microbial mat that contains stalked bacteria [209] can be useful for extreme biomimetics. Researchers simply simulated the temperature-pressure conditions of diagenesis in the laboratory. It was shown that unique mineral structures appear on stalks mainly composed of long-chain saturated aliphatic compounds as temperature and pressure conditions were increased to 250 °C and 140 MPa. Fe minerals, as they transform to stable crystalline phases, probably act as physical protection for the biopolymer-based twisted matrix and help preserve the main organic components under diagenetic conditions [209]. These experiments open the way for extreme biomimetics to design novel iron-containing composite materials [210] simulating polyextremophilic conditions.

## 10. Tellurium Biomineralization

**Figure 5:**
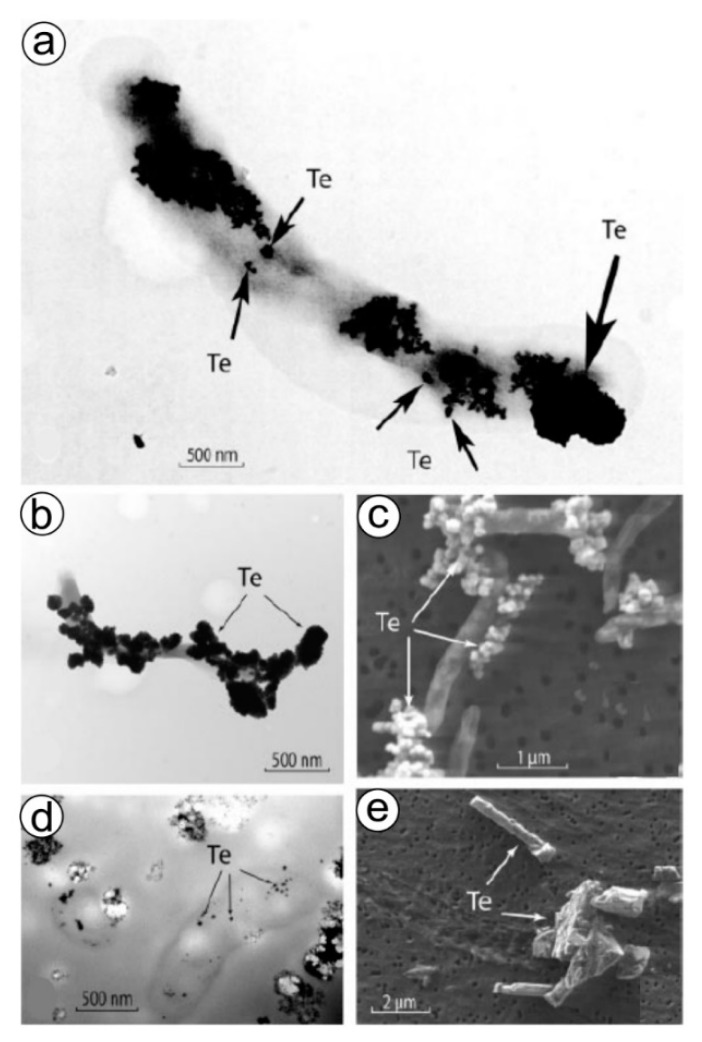
Formation of irregular Te(0) “nanospheres” by *Sulfurospirillum barnesii.* (**a**) Whole-mount TEM image of a single cell grown on Te(VI), showing abundant external Te nanospheres (small arrows) forming larger aggregates (large arrow) on the cell surfaces. (**b**) Lower-magnification TEM image of another single cell grown on Te(VI) showing abundant external Te nanospheres. (**c**) Wide-field SEM image of the Te(0) nanosphere aggregates formed after growth on Te(VI). (**d**) Unstained TEM thin-section image of cells grown on Te(VI) showing internal accumulations of Te(0). (**e**) SEM image of Te(0) obtained from a chemical supply house. Reproduced from [211] with permission from the American Society for Microbiology.

The anaerobic formation of tellurium-based nanostructured biominerals (Figure 5) is an additional example of forced biomineralization (see section above) and seems to be one of the detoxification mechanisms used for dealing with Te-ions. It was clearly shown [211] that the anaerobic growth of *Bacillus selenitireducens* and *Sulfurospirillum barnesii* can be achieved by employing tellurium oxyanions Te(IV) and Te(VI) as electron acceptors (Figure 5). Dissimilatory reduction of Te oxyanions by both microorganisms results in the formation of unusual Te(0) crystals with different structures and nanomorphologies that can occur internally but mainly externally. Those synthesized by *B. selenitireducens* initially are nanorods (10 nm diameter and 200 nm length), which cluster together, forming larger rosettes (about 1000 nm) composed of numerous individual shards [211]. However, *S. barnesii* forms mostly irregularly shaped nanospheres (diameter < 50 nm) that coalesce into larger composite aggregates. The presence of some organic templates within these Te-containing biominerals is still unknown. However, preliminary analytical measurements revealed the presence of functional amide groups on the Te(0), suggesting that some cell wall proteins remained firmly attached to the Te(0) even after being subjected to our purification steps [211].

We suggest that the unexplored phenomena of anaerobic biomineralization have high future potential with respect to the biogenic synthesis of metallic nanoparticles using selected anaerobic microorganisms. Biosynthesis of Te-nanoparticles has also been observed in select marine bacteria [212]. TeO needle-like particles (20–465 nm) have been recently reported as a result of TeO_3_^2−^ exposure in the culture medium of *Phanerochaete chrysosporium* fungus [213] that is able to reduce tellurite (TeO_3_^2−^) to TeO.

## 11. Acidophilic Biomineralization as an Example of Forced Biomineralization

Acidic sulfate and chloride environments often contain Fe(II), As(III), and S(-II), providing several electron donors for chemolithotrophic metabolism [214,215]. As a result, the biomineralization of Fe(III) solid phases are a common occurrence; microbial cells also serve as nucleation sites for the oxidation and or precipitation of Fe(III) minerals [216], including Schwertmannite (Fe_8_O_8_(OH)_6_(SO_4_) *n* H_2_O) [217,218,219] and magnetite (Fe_3_O_4_). It has been detected that both archaeal-bacterial and fungal members of extreme ecosystems were shown to play an active role in the formation of stalactites [220]. Extreme acidophilic organisms have an optimum pH of <3; some of them are even able to live at pH ~1 [214]. All modern organisms must control their intracellular metal and metalloid concentrations, as some metals are essential micronutrients acting in processes such as electron transport. In contrast, other metals such as As, Cd, Au, U, [215], and Hg have no known biological function and are toxic at high concentrations. Therefore, acidophilic organisms have developed a range of uptake and resistance strategies to maintain intracellular metals at desired concentrations [216,217]. Acidophilic microorganisms, defined as having an optimum growth pH of <5, are present in all three domains of life. 

As an example of extreme biomineralization under acidic environmental conditions, special consideration should be paid to microorganisms from Río Tinto in Spain, which hosts an extreme aquatic environment with a remarkably constant acidic pH and a high concentration of heavy metals (Fe, Cu, Zn, As, etc.) [221]. The combined use of conventional and molecular microbial ecology methodologies has shown that 80% of Tinto basin prokaryotic microorganisms correspond to microorganisms belonging to three bacterial genera: *Acidithiobacillus, Leptospirillum,* and *Acidiphilium,* all members of the iron cycle. All *Leptospirillum* spp. isolated from Río Tinto are aerobic iron oxidizers. On the other hand, it has been observed that eukaryotic microorganisms contribute over 60% of the Tinto basin biomass [219]. Acidophilic organisms contribute to the precipitation of amorphous iron oxyhydroxides or siderite (FeCO_3_) [222] in the modern sediments of the river. It confirms that the presence of biological nucleation sites (cell walls of bacteria or fungi) can modify the expected mineral precipitation schemes offered by the bulk physicochemical conditions in which microorganisms grow. Interestingly, several *Leptospirillum* bacteria species [223] and fungal species *Purpureocillium lilacinum* [224,225] contribute to the formation of jarosite (KFe^3+^_3_(OH)_6_(SO_4_)_2_). It has been found that this mineral preferentially nucleates on the fungal cell wall, even on dead cells, and the extracellular polymeric substances (EPS) released by the microorganisms can serve as nucleation sites for this biomineralization process. Results of experimental studies performed by Oggerin and co-workers [225] prove that the concentration of ferric iron, the ratio between Fe^3+^/Fe^2+^, and the presence and amount of nucleation sites are critical factors for the precipitation of jarosite, although the presence of nucleation sites by themselves is not sufficient to promote jarosite formation. However, the detailed mechanism that these organisms use to saturate hydronium-jarosite but not goethite or hematite, the minerals expected to precipitate due to an increase in the pH, is still unknown [224,225].

Understanding of the mechanisms responsible for the biologically induced formation of minerals by acidophilic organisms might be a new direction in the engineering of biominerals for advanced purposes, including geometrically frustrated magnets [226,227] or additives for building materials [228]. It is worth noting that the similarities between the vast sulfate and iron oxide deposits on Mars and the main sulfide bioleaching products found in the Tinto basin have given Río Tinto the status of a geochemical and mineralogical terrestrial Mars analog [221]. Endolithic environments, the pore space of rocks, is a ubiquitous habitat for acidophilic microorganisms on the Earth and is an important target of the search for life elsewhere in the Solar System [214]. Thus, the deep understanding of biomineralization pathways in acidophilic organisms will have major implications for understanding ancient mineral formation on Earth or extraterrestrial planets.

## 12. Prospects for Practical Use

Approaches inspired by forced biomineralization for metal nanoparticle synthesis have been suggested as valuable alternatives to chemical methods. Synthesis and assembly of metal nanoparticles using biological systems is relatively clean, non-toxic, and environmentally friendly and is thus aligned with green chemistry and sustainable materials engineering and development concepts [229,230,231,232]. Therefore, the increasing interest in biological systems for inspiration and using microorganisms as “workers” in the so-called “living factory” for the production of new functional nanomaterials is observed [230]. Weghuis [233,234] described the large-scale utilization of microorganisms in the production of Bioscorodite at 70 °C as a highly efficient and cost-saving method for arsenic remediation and detoxification. Suresh and co-workers used the advantage of forced biomineralization and reported biofabrication of discrete Au [35] and Ag [235] nanocrystalites using *Shewanella oneidensis* metal-reducing bacteria. Both nanoparticles show properties that can be attractive for biomedical applications. Hennebel et al. [236] used fermentatively cultivated bacteria in the formation of highly active, nanoparticulated Pd catalysts for diatrizoate removal. On the other hand, Coker et al. [237] developed nanoscale ferrimagnetic material (with enhanced magnetic properties) using the Fe(III)-reducing bacterium *Geobacter sulfurreducens* and substitution of Fe ions with Co. Li et al. [238] synthesized novel electrochemical materials with enhanced capacitance and cycling stability using a fungal Mn biomineralization process. Furthermore, microbially-induced calcium carbonate precipitation has been shown to have potential as a remediation strategy for toxic metals such as As, Pb, Cd, Cr, and Cu, since these toxic metals can also be precipitated as insoluble carbonates of biological origin [239].

These examples strongly illustrate how syntheses inspired by forced biomineralization might, in the near future, pave the way towards the development of novel generations of various sustainable metal-based materials with advanced applications.

Both extreme biomineralization and extreme biomimetics [240,241,242,243,244,245] represent scientific niches with broad application in industry and in the study of evolutionary biology, studying natural and artificial phenomena that occur “*below the human zone of comfort*” [246]. Both were recently born at the crossroads between such scientific directions and disciplines as prebiotic chemistry, prebiotic mineralogy, the origin and evolution of Life, hydrothermal venting chemistry and biochemistry, astrobiology, cryobiology, and exobiology [247]. To delve into these research fields, radical thinking must explore unusual and very unique biomineralogical scenarios. Such studies could lead to a better understanding of:

- The biomineralization of iron-, silica- and calcium-based phases at extreme environmental conditions;
- The survival strategies of pro- and eukaryotes using protective advantages of biomineralization due to the functionalization of their cell envelopes;
- The mechanisms controlling fossilization, as well as exceptional preservation of organic templates which strongly bind to the mineral surface [248];
- The underlying mechanisms used by diverse extremophiles and polyextremophiles to exhibit extreme cold (cryo-), heat (thermo-), and pressure (piezo-) tolerance.

Here, we have reviewed the biomineralization that occurs in polyextremophiles. These organisms modify their local microenvironment to create appropriate physicochemical conditions for the precipitation of inorganic compounds [239]. Consequently, their survival appears linked to producing unique biominerals under toxic concentrations of metal ions, habituating under complex environmental extremes such as anaerobic, acidic, or thermal-alkaline conditions. Some of these kinds of biominerals are represented in Table 2. The existence of these biominerals is revolutionizing our understanding of the origin and evolution of life under environmental extremes. Moreover, it is providing information on the possible mechanisms of their formation and structural diversity, as well as enabling direct comparison between physico-chemical and molecular records of biominerals that have been produced under ambient and extreme environmental conditions. 

The ability of organisms to engage in biomineralization seems to be associated with a variety of evolutionary advantages. Biomineralization is regarded as an advantageous approach for organisms to become biologically “stealthy” and protect themselves from external damages of diverse origins [79]. Beyond serving as a passive shield or shelter, biomineralized structures provide organisms with the mechanical advantage of a strong lever arm. Darwin’s theory of evolution suggests that the underlying mechanisms behind this change, “*the drivers of early biomineralization*” [80,263], evolved through the selection of the most effective biomineralogical adaptations for their survival in the harsh conditions of the then natural environment. In spite of the suggestion by John Evans that there are over 62 different biominerals on Earth [149], we suggest that this number, taking into account extremophiles (see Table 2), should be significantly higher. In fact, we should probably not attempt to quantify individual pathways for biomineralization, for—as this review has demonstrated—various extreme water chemistries have provided many uncountable environments for biomineralization to evolve. 

Highly specialized biomineralizing organisms are currently being considered by a range of researchers to have potential economic use for the biological fabrication of metal nanoparticles, as well as a variety of other nanoorganized composite materials. Moreover, the potential to harness extremophiles’ capabilities to produce various novel materials such as biominerals and concretes holds widespread promise in industrial settings. 

Microorganisms have adapted to resist the ecotoxicological impact of diverse metal ions [264]. It can be expected that each microorganism has its own survival limit falling into extreme conditions with a high, sometimes close to supersaturated, concentration of metal ions. Microorganisms resist, however possible, a stress factor such as a sharp increase in the concentration of ions of the corresponding metal. It should be borne in mind that many microorganisms possess a high level of tolerance to toxic heavy metals, which is much higher than their concentrations in the environment (for details, see Oggerin et al. [265]). Furthermore, there are hundreds of species of fungi identified, which survived during years of copper mining in the extremely acidic and metal-rich region of Rio Tinto in Spain [265].

However, despite the apparent evolutionary advantages addressed in this work, forced biomineralization can still be lethal for microorganisms due to the disruption of cellular membranes [265]) or due to the formation of such mineral phases that may hinder nutrient exchange between the surrounding environment and the cells [133]. In such cases, biominerals have also been observed on the dead cells and disrupted cell walls as encrustations [133]. It has been hypothesized “that the mineral acts as a buffering “rucksack” that eventually kills the bacterium but helps the metabolism of parent and progeny” [76].

We can speak about possible negative aspects with respect to ecology, mostly in cases of metal contamination from human activity. Mining and processing of metal ores can produce areas of high metal concentration in which forced biomineralization is advantageous for life to survive, leading to changes in the local ecology. The increase of heavy metal ions in such locations imposes selection pressures on pre-existing microbiota. Consequently, environmental metal contamination can change the diversity of microbial communities via the domination of metal-resistant species (for example, see [266,267]). As an intriguing example, metal resistance co-occurring with antibiotic resistance has been reported in bacteria isolated from metal-contaminated soils, waters, and sewage [1,75,133,266,268,269,270,271,272,273,274,275,276,277,278,279,280,281,282,283,284,285,286,287,288]. The ecological impacts of these essentially manmade organisms are yet to be understood, although the harm possibly caused by forced biomineralizing organisms might plausibly be overshadowed by the metal-concentrated environments they inhabit.

Concerns about the effects of forced biomineralizers may extend to the medical realm as well. For example, simultaneous antibiotic resistance and metal resistance have been observed in oral bacteria isolated from infected teeth that had metal dental restorations. The common use of metals in medical implants throughout the human body introduces the potential for considerably elevated metal concentrations to occur with reported negative effects of certain metal implants ranging from systemic illness to local reactions to the carcinogenicity of uncertain etiology (in cases of certain implants). Pathogenic forced biomineralizers in the human body could conceivably cause harm, perhaps drug-resistant infections or even inflammation (and possibly cancer) induced by metal nanoparticles. An improved understanding of metal resistance in medical contexts might therefore help to advance the safety of implanted technology.

## 13. Outlook

Biomineralization can be broadly defined as the formation of minerals by life. Much of the scientific focus on biomineralization has centered on a range of siliciclastic and calcium carbonate (CaCO_3_) skeletons evolving during the Cambrian explosion (ca. 541 Ma) and afterward. However, life’s evolutionary progress in developing biologically mediated mineral production is broadly understood to have predated evidence for the emergence of calcium carbonate mineralization. Life is understood to have evolved the capability to manipulate metals and metalloids to produce a variety of metallic minerals. Polysaccharide templates are widely found to be a common tool used by life for bringing about the precipitation of metallic biominerals. This review discusses a large and interdisciplinary body of work addressing the wide range of metal biomineralization pathways found on Earth, particularly those observed in modern extreme metal-rich aqueous environments. Many examples today are found in the wastewater of mines. 

As discussed in this review, a range of interdisciplinary work has shed light on the tendency for extremophiles living in aqueous environments with elevated metal concentrations to concentrate metals into crystals. The metal biomineralization pathways used by metal-tolerant organisms are widely understood to help organisms avoid poisoning as a means of enduring high metal concentrations. Moreover, beyond the context of forced metal biomineralization in heavily metal-contaminated environments, mechanisms allowing metal biomineralization in a range of environments are understood to offer evolutionary advantages beyond regulation of metal concentrations, including functionalities as broad-reaching as indurated structures for predation protection against a wide variety of assaults from the environment, and functionality as compass needles. Additional focus on classifying the fossil record of metallic biominerals is relevant to the study of life’s origin–in particular, the currently popular idea that metal-rich hydrothermal environments–including black smokers on Earth–are broadly considered as potentially favorable spots for life to originate. 

Without a doubt, scientific interest in the genes related to biomineralization (including forced biomineralization) remains in-trend due to their principal regulatory role in the biosynthesis of macromolecules (i.e., polypeptides and proteins) with high templating activity in the formation of biominerals. The genomics of biomineralization has advanced rapidly and is a topic of active work, however mostly in such directions as calcification, biosilicification, and the formation of biomagnetic structures. Thus, genomic architectures within diverse genetic toolkits have been intensively studied in calcifying organisms (i.e., bacteria], corals, sea urchins, hemichordates, mollusks [289], biosilicifiers (i.e., diatoms [290], sponges [18,291,292,293], and especially magnetotactic microorganisms) to carry out the understanding of biomineralization. However, we cannot exclude the occurrence of so-called “gene-independent biomineralization” [294] in the case of forced biomineralization. For example, such phenomena have been reported for some viruses, which are found in a mineralized state inside archaea cells in hot springs.

Genetically-encoded forced biomineralization seems to be an intriguing direction due to the existence of the open question concerning the possible relationship between well-studied metal resistant genes in bacteria [295] and their possible role in the formation of corresponding mineral phases. Furthermore, the development of computationally predicted gene regulatory networks [289] for forced biomineralization seems to be necessary for the near future.

Although the principles that describe metal formation in organisms are outside the scope of this review, we take the liberty to recommend readers to obtain corresponding details from other recently published review papers, where such phenomena as metal-biomolecule affinities [296,297,298,299,300,301], metal-ion binding sites based on amino acid sequences, and cellular dynamics of metal ion-exchange have been excellently represented. Moreover, recent advances in the understanding of mechanisms of metal formation in diverse biological systems are to be found in numerous overviews on metallomics.

In addition to the widely considered biological implications of metal biomineralization pathways, we have discussed a range of literature addressing biomimetic approaches to the use of forced metal biomineralization in the fabrication and remediation of metals in a wide range of economically relevant contexts. Many applications of forced biomineralization harness the ability of organisms to chelate metals–and, accordingly, to lower concentrations of metals in their environments. The ability of forced biomineralizers to remove metals from solution holds wide promise for a range of uses in medical ecology and society. As we have discussed in this review, the use of forced biomineralizers in the remediation of mine waste pools is receiving wide attention–potentially offering hope for reduced environmental impacts and economic costs of mineral extraction. Furthermore, being considered are bioinspired approaches to medical chelation therapy, using the tools developed by metallophiles. Moreover, patents are already underway to potentially revolutionize how arsenic can be removed from drinking water.

Knowledge of extreme biomineralization and forced biomineralization is a driving force toward recent progress in extreme biomimetics, and organisms that undergo forced metal biomineralization offer many potential avenues for applications in biomaterial-inspired chemistry.

## Figures and Tables

**Table 2:** Selected examples of biominerals produced by a variety of forced biomineralization pathways.

Biomineral	Chemical Formula	Organism	Metal/Metalloid	Ref.
Alamosite	PbSiO_3_	*Bacillus* sp. KK1	Pb	[249]
Bioscorodite	FeAsO_4_·2H_2_O	*Acidianus suljidivorans*, *Sulfolobales* spp. (70 °C, pH 1.2)	FeAs	[118,250,251]
Chernikovite	H_2_(UO_2_)_2_(PO_4_)_2_·8H_2_O	*Anabaena torulosa* (cyanobacteria)	U	[118]
Eskaloite	Cr_2_O_3_	*Schwanniomyces occidentalis* (50 °C)	Cr(VI)	[252]
Greigite	Fe_3_S_4_	*Chrysomallon squamiferum* (thermophilic gastropod)	Fe	[253]
Hydro- cerussite	(Pb_3_(CO_3_)_2_(OH)_2_)	*Paecilomyces javanicus*	Pb	[254]
Kutnahorite	(Ca(Mn^2+^,Mg,Fe^2+^)(CO_3_)_2_)	*Idiomarina* sp. (Halophilic)	Ca, Mn, Mg, Fe	[255]
Ni-struvite	Ni(NH_4_)(PO_4_)∙6H_2_O	*Streptomyces acidiscabies*	Ni	[256]
Orpiment	As_2_S_3_	*Shewanella* sp. *Desulfosporosinus auripigmenti* (anaerobic)	As	[257,258]
Otavite	CdCO_3_	*Neurospora crassa* (fungus)	Cd	[259]
Plumbonacrite	(Pb_10_(CO_3_)_6_O(OH)_6_)	*Paecilomyces javanicus*	Pb	[259]
Pyrite	FeS_2_	*Chrysomallon squamiferum* (thermophilic gastropod)	Fe	[260]
Pyromorphite	Pb_5_(PO_4_)_3_Cl	*Paecilomyces javanicus*	Pb	[261]
Realgar	AsS	As-reducing bacterium closely related to *Caloramator* and *Thermobrachium*. (Anaerobic, moderately thermophilic)	As	[262]
Sphalerite/Wurtzite	(Zn_0.88_Fe_0.12_)S	*Alvinella pompejana* (thermophilic worm)	Zn, Fe	[78]
Tellurium-based biominerals	Te(0)	*Sulfurospirillum barnesii* (anaerobic)	Te	[211]

## Data Availability

Not applicable.
